# Supplementary material for: Polynucleotide phosphorylase protects against renal tubular injury via blocking mt-dsRNA-PKR-eIF2α axis
Source: Nat Commun. 2023 Mar 3;14:1223. doi: 10.1038/s41467-023-36664-0 (PMC9984537; doi:10.1038/s41467-023-36664-0)
Supplement: Supplementary file 1 — Supplementary Information [file 41467_2023_36664_MOESM1_ESM.pdf]

Supplementary Data for

Polynucleotide phosphorylase protects against renal tubular injury via blocking mt-dsRNA-PKR-eIF2α axis

Yujie Zhu[1*], Mingchao Zhang[1,2*], Weiran Wang[1*], Shuang Qu[3*], Minghui Liu[3], Weiwei Rong[1], Wenwen Yang[1], Hongwei Liang[3], Limin Li[3¶], Zhihong Liu[2¶], and Ke Zen[1,3¶]

File list

1. Supplementary Table 1
2. Supplementary figure 1
3. Supplementary figure 2
4. Supplementary figure 3
5. Supplementary figure 4
6. Supplementary figure 5
7. Supplementary figure 6
8. Supplementary figure 7
9. Supplementary figure 8

Supplementary Table 1. Oligonucleotide primers used in the study.

**Primer (qRT-PCR)**	**Gene**	**Sequence (5'-3')**
mt-ND6 forward	*mt-ND6*	CCAATAGGATCCTCCCGAAT
mt-ND6 reverse	*mt-ND6*	AGGTAGGATTGGTGCTGTGG
mt-CO1 forward	*mt-CO1*	ACGTTGTAGCCCACTTCCAC
mt-CO1 reverse	*mt-CO1*	TGGCGTAGGTTTGGTCTAGG
mt-ND5 forward	*mt-ND5*	TCGAAACCGCAAACATATCA
mt-ND5 reverse	*mt-ND5*	CAGGCGTTTAATGGGGTTTA
mt-ND4 forward	*mt-ND4*	AACGGATCCACAGCCGTA
mt-ND4 reverse	*mt-ND4*	AGTCCTCGGGCCATGATT
mt-CYB forward	*mt-CYB*	AGACAGTCCCACCCTCACAC
mt-CYB reverse	*mt-CYB*	GGTGATTCCTAGGGGGTTGT
β-actin mRNA forward	*β-actin*	CTGTGGCATCCACGAAACTA
β-actin mRNA reverse	*β-actin*	AGTACTTGCGCTCAGGAGGA

Primer sequences for strand-specific reverse transcription

**Primer**	**Sequences (5′-3′)**
CO1 Heavy	CGCAAATGGGCGGTAGGCGTGGTTGTGATAAGGGTGGAGAGG
CO1 Light	CGCAAATGGGCGGTAGGCGTGTCAAACTCAAACTACGCCCTG
ND4 Heavy	CGCAAATGGGCGGTAGGCGTGTGTTTGTCGTAGGCAGATGG
ND4 Light	CGCAAATGGGCGGTAGGCGTGCCTCACACTCATTCTCAACCC
ND5 Heavy	CGCAAATGGGCGGTAGGCGTGTTTGGGTTGAGGTGATGATG
ND5 Light	CGCAAATGGGCGGTAGGCGTGCATTGTCGCATCCACCTTTA
ND6 Heavy	CGCAAATGGGCGGTAGGCGTGGGTTGAGGTCTTGGTGAGTG
ND6 Light	CGCAAATGGGCGGTAGGCGTGCCCATAATCATACAAAGCCCC
CYB Heavy	CGCAAATGGGCGGTAGGCGTGGGATAGTAATAGGGCAAGGACG
CYB Light	CGCAAATGGGCGGTAGGCGTGCAATTATACCCTAGCCAACCCC
β-actin	CGCAAATGGGCGGTAGGCGTGACA CAG AGTACTTGCGCTCAG

Primer sequences for strand-specific qPCR

**Gene**	**Forward Primer (5′-3′)**	**Reverse Primer (5′-3′)**
CO1 Heavy	TCAAACTCAAACTACGCCCTG	CGCAAATGGGCGGTAGGCGTG
CO1 Light	GTTGTGATAAGGGTGGAGAGG	CGCAAATGGGCGGTAGGCGTG
ND4 Heavy	CTCACACTCATTCTCAACCCC	CGCAAATGGGCGGTAGGCGTG
ND4 Light	TGTTTGTCGTAGGCAGATGG	CGCAAATGGGCGGTAGGCGTG
ND5 Heavy	CTAGGCCTTCTTACGAGCC	CGCAAATGGGCGGTAGGCGTG
ND5 Light	TAGGGAGAGCTGGGTTGTTT	CGCAAATGGGCGGTAGGCGTG
ND6 Heavy	TCATACTCTTTCACCCACAGC	CGCAAATGGGCGGTAGGCGTG
ND6 Light	TGCTGTGGGTGAAAGAGTATG	CGCAAATGGGCGGTAGGCGTG
CYB Heavy	CAATTATACCCTAGCCAACCCC	CGCAAATGGGCGGTAGGCGTG
CYB Light	GGATAGTAATAGGGCAAGGACG	CGCAAATGGGCGGTAGGCGTG
β-actin	ACACAGTGCTGTCTCGTGGTA	CGCAAATGGGCGGTAGGCGTG

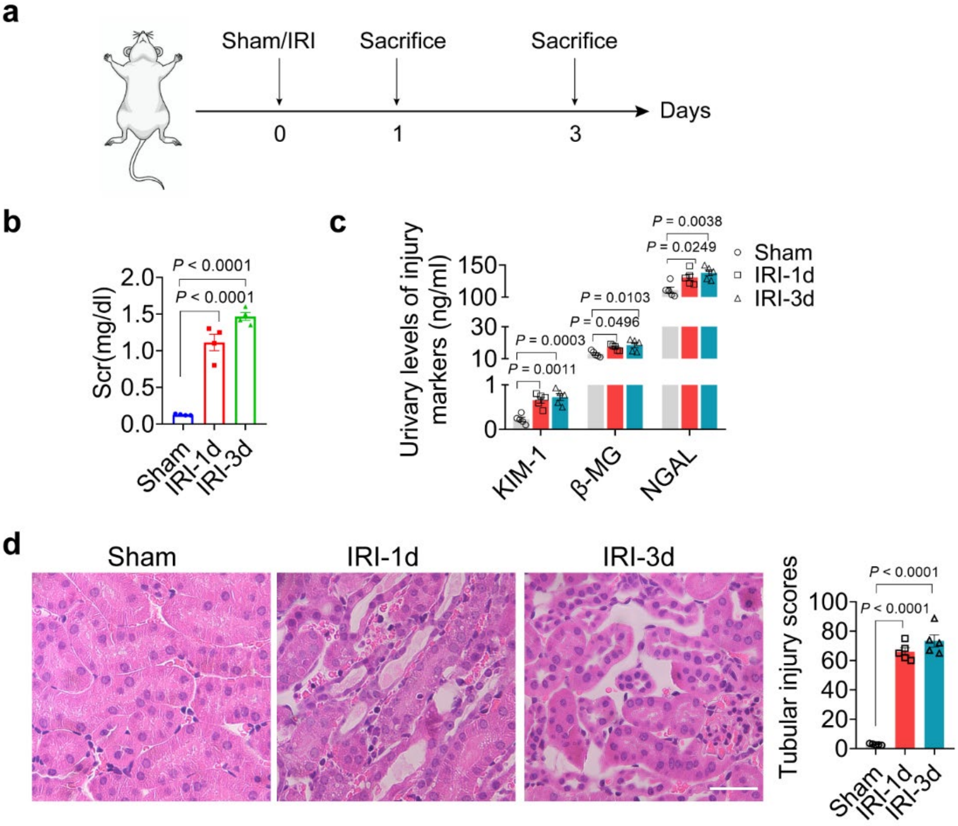

Supplementary figure 1. Establishment of IRI mouse model. **a**, Schematic of IRI mouse model experimental approach. **b**, Scr in WT and IRI mice. **c**, Urinary levels of renal tubular injury markers in WT and IRI mice. **d**, Left: H&E staining of kidney tissue sections from WT and IRI mice. Right: quantification of tubular injury score. n = 5 mice/group, 8w. Scale bar, 50μm. The above experiments were successfully repeated three times. One-way ANOVA with Dunnett's multiple comparisons test was performed in (b, d). One-way ANOVA with Sidak's multiple comparisons test was performed in (c), and the results were presented as mean ± SEM. Image of mouse in (a) was created with BioRender.com. Source data are provided in Source Data file.

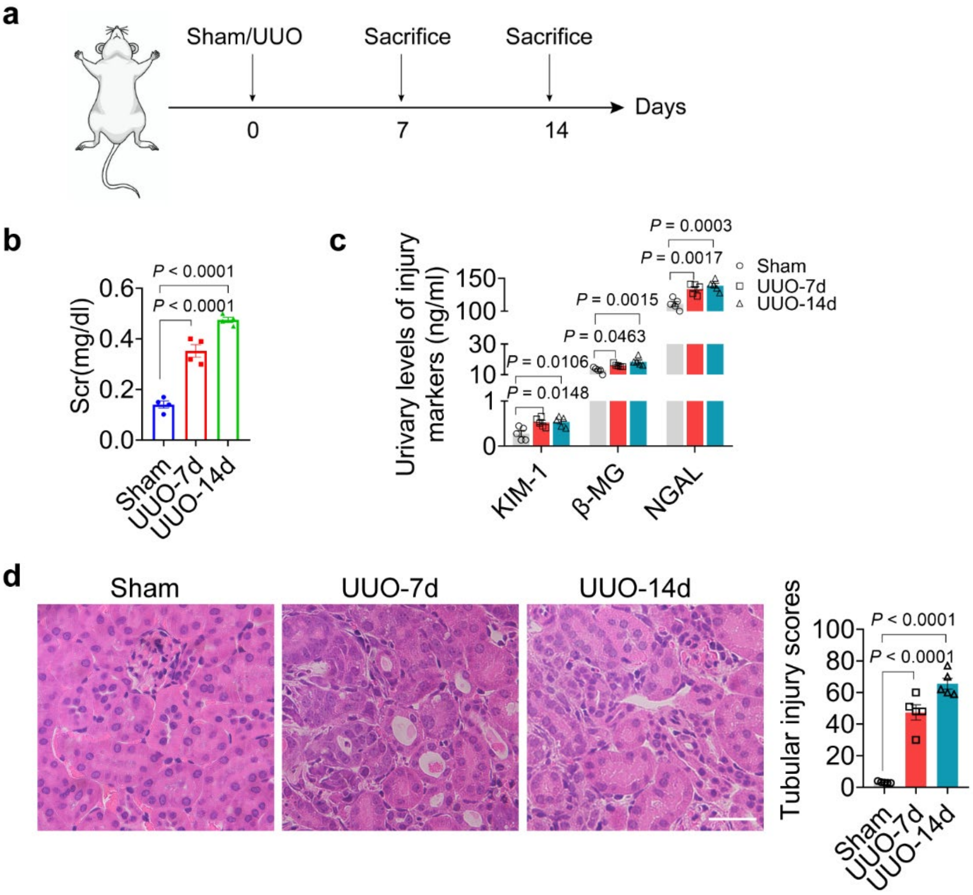

Supplementary figure 2. Establishment of UUO mouse model. **a**, Schematic of UUO mouse model experimental approach. **b**, Scr in WT and UUO mice. **c**, Urinary levels of renal tubular injury markers in WT and UUO mice. **d**, Left: H&E staining of kidney tissue sections from WT and UUO mice. Right: quantification of tubular injury score. n = 5 mice/group, 8w. Scale bar, 50μm. The above experiments were successfully repeated three times. One-way ANOVA with Dunnett's multiple comparisons test was performed in (b, d). One-way ANOVA with Sidak's multiple comparisons test was performed in (c), and the results were presented as mean ± SEM. Image of mouse in (a) was created with BioRender.com. Source data are provided in Source Data file.

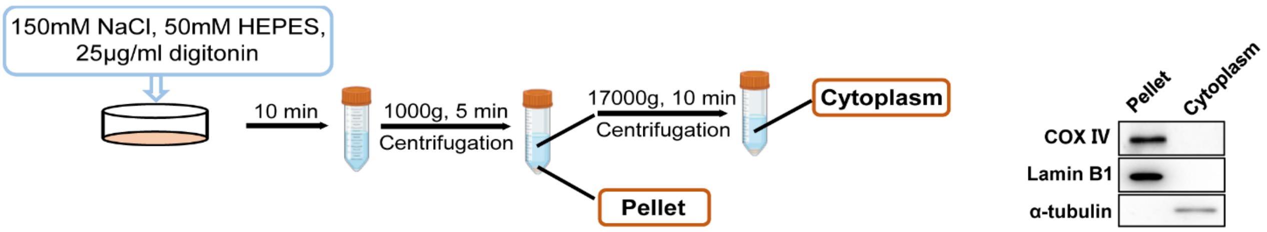

Supplementary figure 3. Isolation and validation of mitochondria-free cytosolic fraction. Left, schematic of experimental approach to isolate mitochondria-free cytosolic fraction from renal tubules or cells. Right, WB validation of isolated cytosolic fraction. Data were from 3 independent experiments. Images of test tubes were created with BioRender.com.

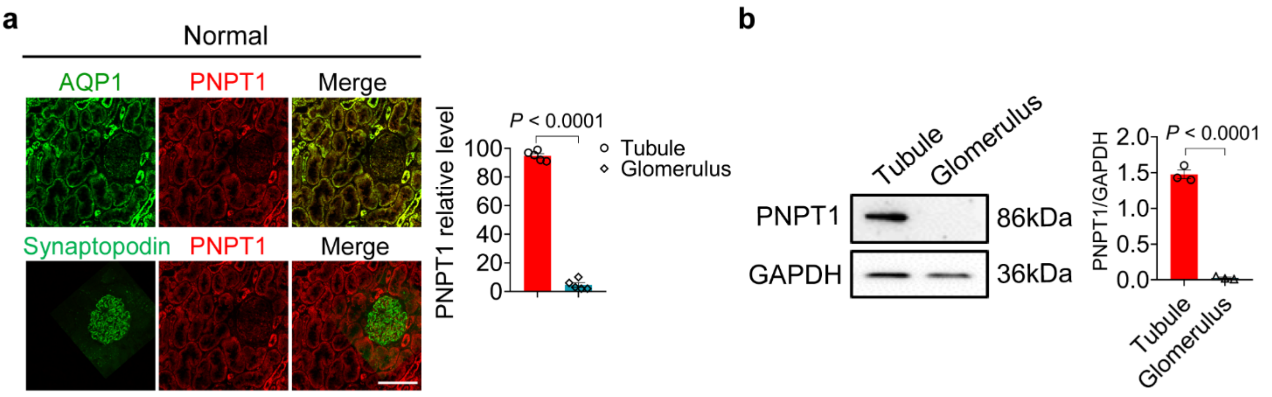

Supplementary figure 4. Expression and distribution of PNPT1 in normal human kidney. a, Left: immunofluorescence labeling of PNPT1 in human renal tubule and glomerulus. Scale bar, 50μm. Right: quantification of PNPT1 level (n = 5); **b**, WB analysis of PNPT1 in human renal tubule and glomerulus (n = 3). The above experiments were successfully repeated three times. Two-tailed unpaired t test was performed for the statistical analyses in (a-b), and the results were presented as the mean ± SEM. Source data are provided in Source Data file.

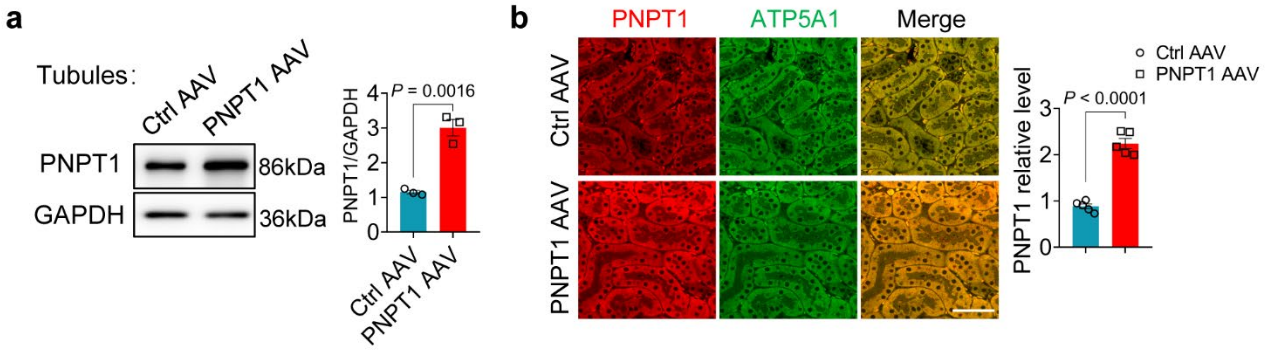

Supplementary figure 5. PNPT1 AAV increased PNPT1 expression in mouse renal tubules. a, WB analysis of PNPT1 in mouse renal tubules after Ctrl AAV or PNPT1 AAV infections (3 mice/group, 8w); **b**, Left: Co-localization of PNPT1 (red) and mitochondria (ATP5A1, green) in mouse renal tubules. Right: quantification of PNPT1 relative level (n=5 mice/group, 8w). Scale bar, 50μm. The above experiments were successfully repeated three times. Two-tailed unpaired t test was performed for the statistical analyses in (a-b), and the results were presented as the mean ± SEM. Source data are provided in Source Data file.

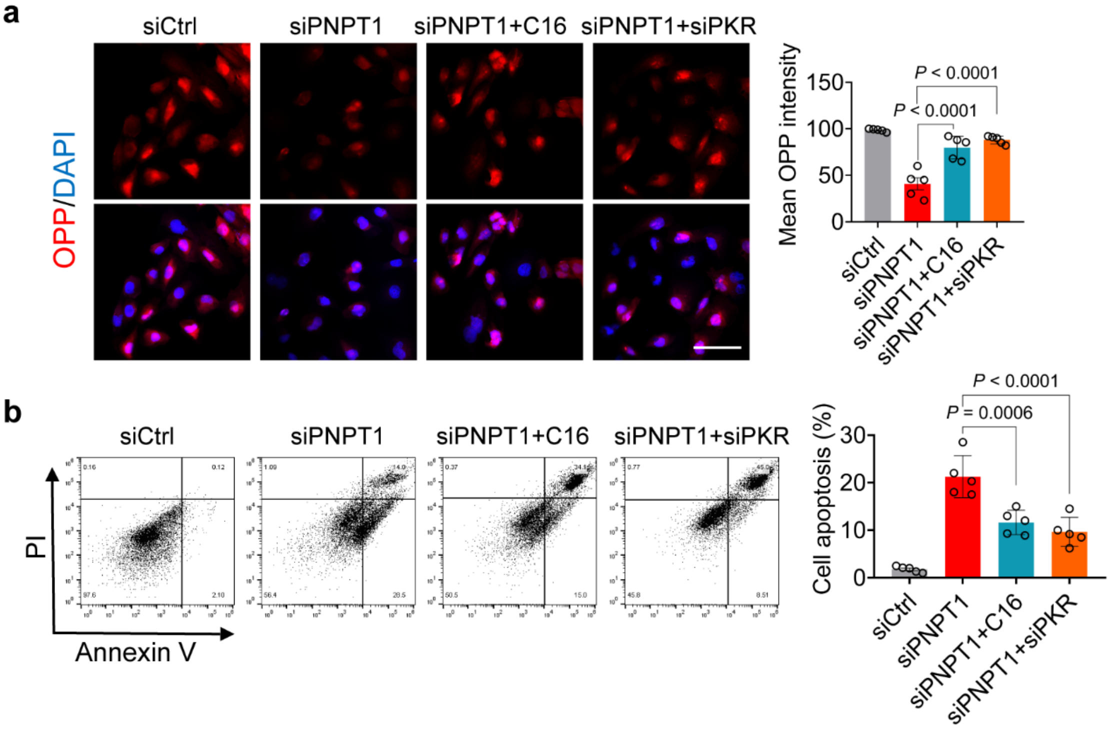

Supplementary figure 6. Knockdown of PKR resulted in a similar phenotype as that of C16 in PNPT1-deficient HK2 cells. a, Left, measurement of total protein synthesis in PNPT1-deficient HK2 cells transfected with siPKR plasmid or treated with C16 using Click-iT® Plus OPP Protein Synthesis Assay Kit. Right: quantification of OPP protein synthesis (n = 5). **b**, Apoptosis of siPNPT1-transfected HK2 cells treated with siPKR plasmid or C16 (n = 5). Scale bar, 50μm. The above experiments were successfully repeated three times. One-way ANOVA with Tukey's multiple comparisons test was performed in (a-b) and the results were presented as mean ± SEM. Source data are provided in Source Data file.

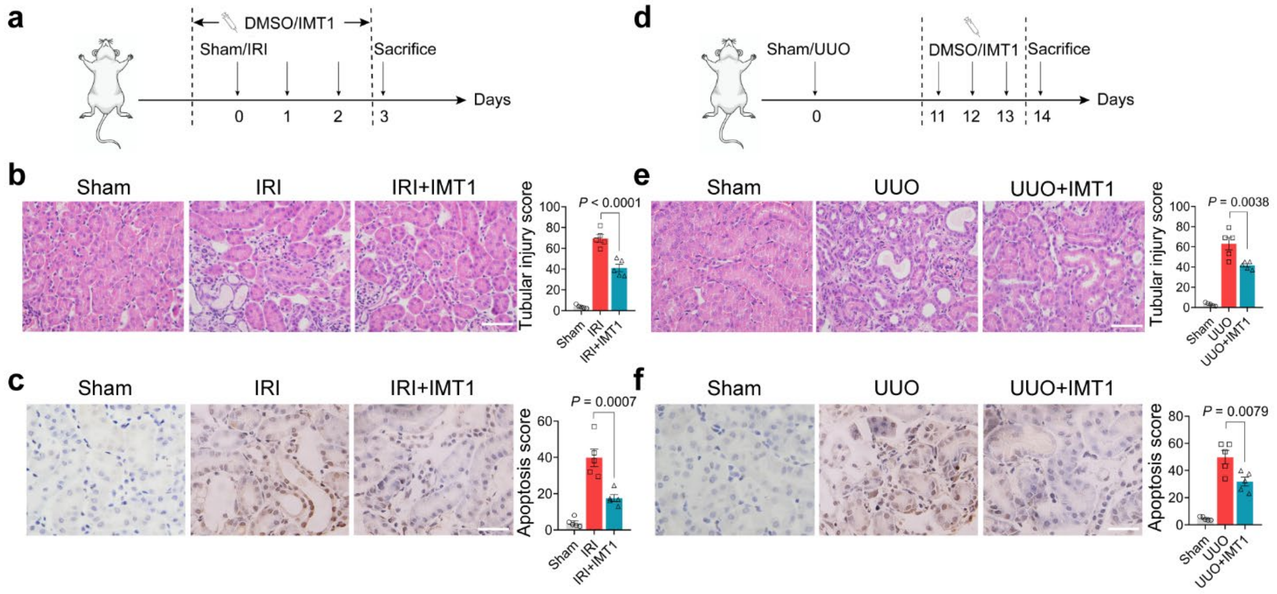

Supplementary figure 7. IMT1 treatment markedly attenuated renal tubular damage induced by IRI or UUO procedure. a, Schematic of experimental approach in IRI mouse model. **b**, Left: H&E staining of kidney tissue sections from IRI mice treated with or without IMT1. Right: quantification of tubular injury score. **c**, Left: TUNEL assay of kidney tissue sections from IRI mice treated with or without IMT1. Right: quantification of apoptosis in kidney tissues. **d**, Schematic of experimental approach in UUO mice. **e**, Left: H&E staining of kidney tissue sections from UUO mice treated with or without IMT1. Right: quantification of tubular injury score. **f**, Left: TUNEL assay of kidney tissue sections from UUO mice treated with or without IMT1. Right: quantification of apoptosis in kidney tissues. n=5 mice/group, 8w. Scale bars, 50μm. The above experiments were successfully repeated three times. One-way ANOVA with Tukey's multiple comparisons test was performed in (b-c, e-f) and the results were presented as mean ± SEM. Images of mouse in (a, d) were created with BioRender.com. Source data are provided in Source Data file.

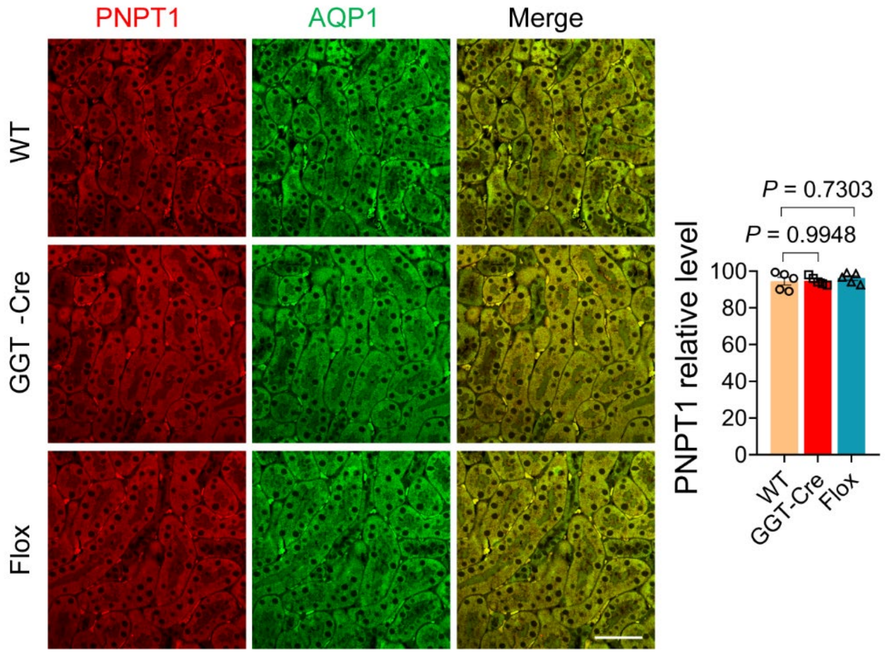

Supplementary figure 8. Immunofluorescence analysis of PNPT1 level and distribution in mouse tubules with GGT-Cre only or floxed only genetic modification. n=5 mice/group, 8w. Scale bar, 50μm. The above experiments were successfully repeated three times. One-way ANOVA with Tukey's multiple comparisons test was performed, and the results were presented as mean ± SEM. Source data are provided in Source Data file.
