# Supplementary material for: Polynucleotide phosphorylase protects against renal tubular injury via blocking mt-dsRNA-PKR-eIF2α axis
Source: Nat Commun. 2023 Mar 3;14:1223. doi: 10.1038/s41467-023-36664-0 (PMC9984537; doi:10.1038/s41467-023-36664-0)
Supplement: Supplementary file 3 — Reporting Summary [file 41467_2023_36664_MOESM3_ESM.pdf]

nature portfolio

Corresponding author(s): Ke Zen

Last updated by author(s): Jan 21, 2023

Reporting Summary

Nature Portfolio wishes to improve the reproducibility of the work that we publish. This form provides structure for consistency and transparency in reporting. For further information on Nature Portfolio policies, see our Editorial Policies and the Editorial Policy Checklist.

Statistics

For all statistical analyses, confirm that the following items are present in the figure legend, table legend, main text, or Methods section.

n/a	Confirmed	
☐	☒	The exact sample size (*n*) for each experimental group/condition, given as a discrete number and unit of measurement
☐	☒	A statement on whether measurements were taken from distinct samples or whether the same sample was measured repeatedly
☐	☒	The statistical test(s) used AND whether they are one- or two-sided<br>*Only common tests should be described solely by name; describe more complex techniques in the Methods section.*
☒	☐	A description of all covariates tested
☐	☒	A description of any assumptions or corrections, such as tests of normality and adjustment for multiple comparisons
☐	☒	A full description of the statistical parameters including central tendency (e.g. means) or other basic estimates (e.g. regression coefficient) AND variation (e.g. standard deviation) or associated estimates of uncertainty (e.g. confidence intervals)
☐	☒	For null hypothesis testing, the test statistic (e.g. *F*, *t*, *r*) with confidence intervals, effect sizes, degrees of freedom and *P* value noted<br>*Give P values as exact values whenever suitable.*
☒	☐	For Bayesian analysis, information on the choice of priors and Markov chain Monte Carlo settings
☒	☐	For hierarchical and complex designs, identification of the appropriate level for tests and full reporting of outcomes
☒	☐	Estimates of effect sizes (e.g. Cohen's *d*, Pearson's *r*), indicating how they were calculated

Our web collection on statistics for biologists contains articles on many of the points above.

Software and code

Policy information about availability of computer code

Data collection	All fluorescence images were captured on a confocal microscope (Zeiss LSM 880) with ZEN 3.1 blue edition software. Histological and immunohistochemistry analysis were captured by microscopy (Olympus BX53). Flow cytometry was carried out on a flow cytometer (Thermo Fisher Scientific) . Bone mineral density (BMD) was evaluated by Ultra Focus DXA (Faxitron). Electron micrographs were obtained using a Hitachi 7500 transmission electron microscope.
Data analysis	For quantification of western blots and all images, we used ImageJ 1.44p software. Flow cytometry was analysed by FlowJo V10.4 software. For statistical analyses, we used GraphPad PRISM 8 software.

For manuscripts utilizing custom algorithms or software that are central to the research but not yet described in published literature, software must be made available to editors and reviewers. We strongly encourage code deposition in a community repository (e.g. GitHub). See the Nature Portfolio guidelines for submitting code & software for further information.

Data

Policy information about availability of data

All manuscripts must include a data availability statement. This statement should provide the following information, where applicable:

- Accession codes, unique identifiers, or web links for publicly available datasets
- A description of any restrictions on data availability
- For clinical datasets or third party data, please ensure that the statement adheres to our policy

All relevant data generated for this study are included in the article/Supplementary Material/Source Data File. Source data were provided with this paper.

Human research participants

Policy information about studies involving human research participants and Sex and Gender in Research.

Reporting on sex and gender	For human kidney tissue, sample numbers of male or female participants were indicated.
Population characteristics	There is no population characteristics analysis in the present manuscript. Selection of age and gender are random during sample collection. ATN (n=12, 5 males and 7 females), DN (n=14, 8 males and 6 females), IgAN (n=16, 9 males and 7 females), LN (n=15, 5 males and 10 females), MN (n=13, 8 males and 5 females) and FSGS (n=15, 6 males and 9 females), as well as 5 non-renal tubular injury controls (3 males and 2 females).
Recruitment	Human kidney tissues were randomly collected. There is no potential self-selection bias or other biases that could affect the results.
Ethics oversight	All protocols concerning the use of patient samples in this study were approved by the Human Subjects Committee of Jinling Hospital, Nanjing University School of Medicine (2019NZKYKS-008-01).

Note that full information on the approval of the study protocol must also be provided in the manuscript.

Field-specific reporting

Please select the one below that is the best fit for your research. If you are not sure, read the appropriate sections before making your selection.

- [x] Life sciences
- [ ] Behavioural & social sciences
- [ ] Ecological, evolutionary & environmental sciences

For a reference copy of the document with all sections, see nature.com/documents/nr-reporting-summary-flat.pdf

Life sciences study design

All studies must disclose on these points even when the disclosure is negative.

Sample size	Sample size base on the data in our previous study and a given power (>0.8) using G-power software 3.1 according to Ding X. et al. Nature communications, 2021. The sample sizes were sufficient for statistical analysis.
Data exclusions	No data were excluded from the analyses.
Replication	The experimental findings were reproduced in multiple independent experiments. The number of independent experiments and biological replicates in each data panel is indicated in the figure legends.
Randomization	Mice/cells were assigned randomly into experimental groups and processed in an arbitrary order. Patients were diagnosed and grouped based on renal biopsies at the National Clinical Research Center of Kidney Diseases, Jinling Hospital, Nanjing University School of Medicine.
Blinding	All the enrolled mice or subsequent samples were labelled only with mouse ID numbers and did not indicate genotype or type of treatment. Genotype or treatment type were decoded after the data acquisition and quantification analysis were complete.

Reporting for specific materials, systems and methods

We require information from authors about some types of materials, experimental systems and methods used in many studies. Here, indicate whether each material, system or method listed is relevant to your study. If you are not sure if a list item applies to your research, read the appropriate section before selecting a response.

Materials & experimental systems

n/a	Involved in the study
☐	☒ Antibodies
☐	☒ Eukaryotic cell lines
☒	☐ Palaeontology and archaeology
☐	☒ Animals and other organisms
☒	☐ Clinical data
☒	☐ Dual use research of concern

Methods

n/a	Involved in the study
☒	☐ ChIP-seq
☐	☒ Flow cytometry
☒	☐ MRI-based neuroimaging

Antibodies

Antibodies used

dsRNA mAb J2, 1:200, Scions, Cat# 10010200, RRID:AB_2651015
PNPT1 (immunofluorescence), 1:200, Proteintech Cat# 14487-1-AP, RRID:AB_2165820
PNPT1 (western blot), 1:2000, Proteintech Cat# 14487-1-AP, RRID:AB_2165820
AQP1, 1:200, Abcam Cat# ab168387, RRID:AB_2810992
synaptopodin, 1:100, Abcam Cat# ab224491,
podocin, 1:100, Sigma-Aldrich Cat# P0372, RRID:AB_261982
PKR, 1:1000, Abcam Cat# ab32506, RRID:AB_777306
p-PKR, 1:1000, Abcam Cat# ab32036, RRID:AB_777310
eIF2α, 1:1000, Cell Signaling Technology Cat# 5324, RRID:AB_10692650
p-eIF2α, 1:1000, Cell Signaling Technology Cat# 3398, RRID:AB_2096481
ATF4, 1:1000, Abcam Cat# ab184909, RRID:AB_2819059
COX IV, 1:1000, Abcam Cat# ab14744, RRID:AB_301443
LaminB1, 1:1000, Abcam Cat# ab65986, RRID:AB_1140888
α-tubulin, 1:2000, Proteintech Cat# 11224-1-AP, RRID:AB_2210206
goat anti-mouse Alexa Fluor 488, 1:1000, Thermo Fisher Scientific Cat# A-11001, RRID:AB_2534069
donkey anti-rabbit Alexa Fluor 594, 1:1000, Thermo Fisher Scientific Cat# A-21207, RRID:AB_141637
donkey anti-rabbit Alexa Fluor 488, 1:1000, Thermo Fisher Scientific Cat# A-21206, RRID:AB_2535792
goat anti-mouse Alexa Fluor 594, 1:1000, Thermo Fisher Scientific Cat# A-11005, RRID:AB_2534073
goat anti-mouse IgG-HRP, 1:1000, Santa Cruz Biotechnology Cat# sc-2005, RRID:AB_631736
goat anti-rabbit IgG-HRP, 1:1000, Santa Cruz Biotechnology Cat# sc-2004, RRID:AB_631746

Validation

All antibodies have been tested for reactivity against the appropriate species on the specification sheets on the providers' websites or in published articles. A full reference list can be found on the official website of the manufacturer.
According to the manufacturer's website, dsRNA mAb (Scions, 10010500) is suitable for ELISA, dsRNA-immunoblotting, immunoaffinity chromatography and in certain systems also for immunohistochemistry. This antibody was previously validated and data shown in PubMed: 2057357.
According to the manufacturer's website, PNPT1 (Proteintech, 14487-1-AP) is suitable for IF, IHC, IP, WB and ELISA, and reacts with mouse and human. This antibody was previously validated and data shown in PubMed: 23084290.
According to the manufacturer's website, AQP1 (Abcam, ab168387) is suitable for WB, IHC-P, Flow Cyt (Intra), ICC/IF, and reacts with mouse, rat and human. This antibody was previously validated and data shown in PubMed: 33907568.
According to the manufacturer's website, synaptopodin (Abcam, ab224491) is suitable for ICC/IF, WB, IHC-P, and reacts with human. This antibody was previously validated and data shown in PubMed: 31371698.
According to the manufacturer's website, podocin (Sigma-Aldrich, P0372) is suitable for IF, WB CL, and reacts with mouse, rat and human. This antibody was previously validated and functional tested shown on the website.
According to the manufacturer's website, PKR (Abcam, ab32506) is suitable for Flow Cyt (Intra), WB, IHC-P, ICC/IF, IP, and reacts with human. This antibody was previously validated and data shown in PubMed: 33657363.
According to the manufacturer's website, p-PKR (Abcam, ab32036) is suitable for Flow Cyt (Intra), IHC-P, WB, IP, and reacts with human and pig. This antibody was previously validated and data shown in PubMed: 33110236.
According to the manufacturer's website, eIF2α (CST, 5324S) is suitable for IHC-P, WB, IP, and reacts with human, mouse, rat and monkey. This antibody was previously validated and data shown in PubMed: 35859520.
According to the manufacturer's website, p-eIF2α (CST, 3398S) is suitable for IHC-P, WB, IP, and reacts with human, mouse, rat, monkey and fly Drosophila melanogaster. This antibody was previously validated and data shown in PubMed: 36273647.
According to the manufacturer's website, p-eIF2α (CST, 3398S) is suitable for flow Cyt (Intra), WB, IHC-P, ICC/IF, IP, and reacts with human. This antibody was previously validated and data shown in PubMed: 36273647.
According to the manufacturer's website, ATF4 (ab184909) is suitable for flow Cyt (Intra), WB, IHC-P, ICC/IF, IP, and reacts with human. This antibody was previously validated and data shown in PubMed: 33341442.
According to the manufacturer's website, COX IV(Abcam, ab14744) is suitable for Flow Cyt, WB, and reacts with mouse, rat, cow, human. This antibody was previously validated and data shown in PubMed: 33231680.
According to the manufacturer's website, LaminB1 (Abcam, ab65986) is suitable for IHC-Fr, WB, IHC-P, ICC and reacts with rat, human. This antibody was previously validated and data shown in PubMed: 33065093.
According to the manufacturer's website, α-tubulin (Proteintech, 11224-1-AP) is suitable for FC, IF, IHC, IP, WB, ELISA and reacts with human, mouse, rat. This antibody was previously validated and data shown in PubMed: 29643511.

Eukaryotic cell lines

Policy information about cell lines and Sex and Gender in Research

Cell line source(s)	*State the source of each cell line used and the sex of all primary cell lines and cells derived from human participants or vertebrate models.*
Authentication	*Describe the authentication procedures for each cell line used OR declare that none of the cell lines used were authenticated.*
Mycoplasma contamination	*Confirm that all cell lines tested negative for mycoplasma contamination OR describe the results of the testing for mycoplasma contamination OR declare that the cell lines were not tested for mycoplasma contamination.*
Commonly misidentified lines (See ICLAC register)	*Name any commonly misidentified cell lines used in the study and provide a rationale for their use.*

Animals and other research organisms

Policy information about studies involving animals; ARRIVE guidelines recommended for reporting animal research, and Sex and Gender in Research

Laboratory animals	Male C57BL/6J (8w, 22–25 g) and renal tubular cell-specific PNPT1-knockout (KO) mice(1-17w) were obtained from the Model Animal Research Center of Nanjing University. In brief, PNPT1-flox homozygous were first generated using the CRISPR/Cas9 system, and PNPT1-KO mice were obtained by mating PNPT1-flox homozygous and Ggt-Cre mice. All mice were backcrossed to a C57BL/6 background.
Wild animals	none
Reporting on sex	All animals used in this study are male.
Field-collected samples	none
Ethics oversight	All experimental procedures were approved by Model Animal Research Center of Nanjing University and according with Laboratory Animal Care Guidelines.

Note that full information on the approval of the study protocol must also be provided in the manuscript.

Flow Cytometry

Plots

Confirm that:

- [x] The axis labels state the marker and fluorochrome used (e.g. CD4-FITC).
- [x] The axis scales are clearly visible. Include numbers along axes only for bottom left plot of group (a 'group' is an analysis of identical markers).
- [x] All plots are contour plots with outliers or pseudocolor plots.
- [x] A numerical value for number of cells or percentage (with statistics) is provided.

Methodology

Sample preparation	Briefly, cells were resuspended in 0.5 ml binding buffer and incubated with FITC-Annexin V and PI for 15 min in the dark.
Instrument	a flow cytometer (Thermo Fisher Scientific, Attune NxT)
Software	FlowJo
Cell population abundance	No cells were sorted from cell population and no post-sort analysis was done. Cell clumps and debris were excluded.
Gating strategy	Set negative by unstained cells (Figure4e).

- [x] Tick this box to confirm that a figure exemplifying the gating strategy is provided in the Supplementary Information.
